# The Role of Circulating Tumor Cells in Ovarian Cancer Dissemination

**DOI:** 10.3390/cancers14246030

**Published:** 2022-12-07

**Authors:** Anna Szczerba, Aleksandra Śliwa, Pawel P. Pieta, Anna Jankowska

**Affiliations:** 1 Chair and Department of Cell Biology, Poznan University of Medical Sciences, Rokietnicka 5D, 60-806 Poznan, Poland; 2 Department of Bionic and Experimental Medical Biology, Poznan University of Medical Sciences, 60-806 Poznan, Poland

**Keywords:** ovarian cancer, invasiveness, metastasis, CTC, CSC, EMT

## Abstract

**Simple Summary:**

Ovarian cancer is the most lethal type of gynecological cancer. The leading cause of ovarian cancer patients’ death is late diagnosis, disease progression, and metastasis, defined by the spread of invasive cancer cells. About 80% of ovarian cancer patients have disseminated disease at the time of diagnosis. Ovarian cancer metastasis can occur via the transcoelomic, hematogenous, or lymphatic route. Understanding the mechanisms that drive the process of cancer cell dissemination is the key to the development and successful implementation of new diagnostic and treatment methods, particularly drugs and/or therapies targeting metastasis. This review describes ovarian cancer cell dissemination linked to circulating tumor cells (CTCs), with special emphasis on cell biology and their clinical significance.

**Abstract:**

Metastatic ovarian cancer is the main reason for treatment failures and consequent deaths. Ovarian cancer is predisposed to intraperitoneal dissemination. In comparison to the transcoelomic route, distant metastasis via lymph vessels and blood is less common. The mechanisms related to these two modes of cancer spread are poorly understood. Nevertheless, the presence of tumor cells circulating in the blood of OC patients is a well-established phenomenon confirming the significant role of lymphatic and hematogenous metastasis. Thus, the detection of CTCs may provide a minimally invasive tool for the identification of ovarian cancer, monitoring disease progression, and treatment effectiveness. This review focuses on the biology of ovarian CTCs and the role they may play in cancer diagnosis and therapy.

## 1. Introduction

Despite the advance in diagnostic and treatment methods, ovarian cancer (OC) remains the most lethal type among all gynecological cancers [1,2]. 

The leading causes of treatment failures and consequent deaths of ovarian cancer patients are late diagnosis, disease progression, and metastasis, defined by the spread of invasive cancer cells. Dissemination of ovarian cancer is one of its characteristic features; about 80% of ovarian cancer patients have disseminated disease at the time of diagnosis [3]. Even though metastasis is the leading cause of ovarian cancer-related fatalities, our understanding of the mechanisms that regulate the process remains limited.

**Figure 1:**
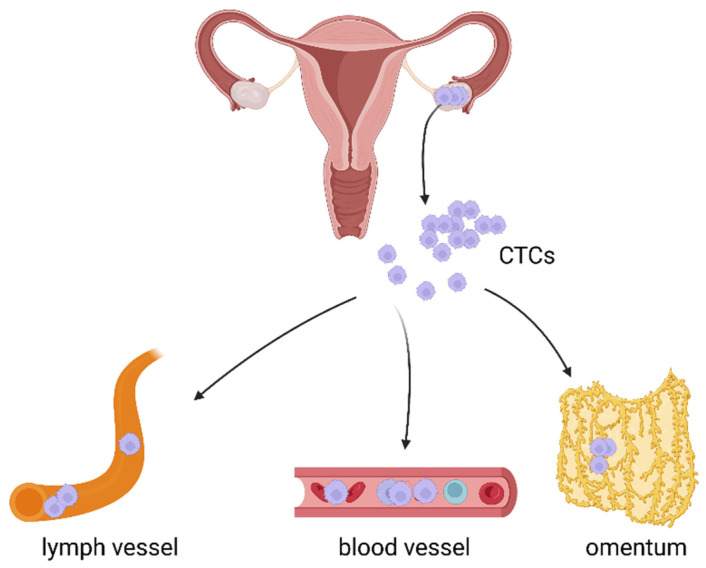
Three main routes of cancer cell dissemination: transcoelomic, hematogenous, and lymphatic. Created with https://biorender.com/, (accessed on 31 October 2022).

Ovarian cancer cells can spread via three main routes: transcoelomic, hematogenous, and lymphatic (Figure 1).

The most common and best known route of OC spread is the transcoelomic route. It is associated with metastasis within the peritoneal cavity and affects the surrounding peritoneal organs [4,5]. In this type of cancer, the dissemination of single cells, multicellular aggregates, and spheroids seed into the mesothelial layer and organs of the peritoneal cavity [6].

Compared to the transcoelomic route, distant metastasis via lymph vessels and blood is less common and the mechanisms related to these two modes of cancer dissemination are poorly understood, and merit detailed investigations.

However, numerous studies confirm that lymphatic and haematogenous spread of ovarian cancer is associated with the presence of cells that can detach from the tumor mass and persist in biological fluids, mainly in blood [7,8,9,10,11,12,13,14,15,16,17]. These cells are known as circulating tumor cells—CTCs.

CTCs, believed to be responsible for the spread of the cancer to lymph nodes and distant organs, can be detected at all stages of ovarian cancer and are known to play a significant role in the disease progression. This review focuses on the biology of circulating tumor cells and the role they play in the hematogenous metastasis of ovarian cancer.

## 2. Biology of Circulating Tumor Cells

Tumor cells circulating in the bloodstream of cancer patients are thought to have the potential to reach and settle in new niches and develop metastasis [11,18]. Thus, their presence, which shows tumor dissemination from the primary site to distant organs, might be an indicator of the disease progression.

The significance of CTCs and hematogenous spread in ovarian cancer is just starting to be recognized. One reason for such negligence is the lack of easily available models of vascular ovarian cancer metastasis. The mechanisms of haematogenous metastasis are studied using a few animal models, including the parabiosis model, traditional murine xenograft models, genetically modified mouse models, as well as in vitro experiments: 3D spheroids, and organoids [13,19]. However, a growing body of research suggests that CTCs play an important role in ovarian cancer metastasis [7,8,9,10,11,12,13,14,15,16,17].

The results of CTCs studies rely on the accessibility of CTCs detection methods. The difficulty in detecting and isolating rare and heterogeneous CTCs in ovarian cancer therefore remains the main limitation. In fact, depending on the techniques used for the CTCs detection, the positivity rates documented in different studies varied from 12% to 90%. The detection rate might be even higher and reach 95%, as it was recently presented using the subtraction enrichment of the cells followed by immunostaining and fluorescence in situ hybridization [20]. It points to the importance of proper isolation methods allowing successful evaluation of ovarian CTCs.

CTCs detection and identification in blood of OC patients is usually based on cell population enrichment using different biomarkers, followed by CTCs molecular profiling. The most popular approaches include: (i) PCR-based methods analyzing tumor-specific transcripts, (ii) immunological assays using monoclonal antibodies specific for tumor (usually epithelial) markers, (iii) isolation by the size of the tumor cells [21].

Markers used in the identification of CTCs include epithelial antigens (EpCAM, WT1, MUC16, MUC1, KRT7, KRT18, and KRT19), mesenchymal and EMT-related factors (vimentin, N-cadherin, Snai2, CD117, CD146, and PI3Kα, Akt-2, TIMP1, CXCR4, and Twist) as well as stem cell markers (CD44, ALDH1A1, Oct4, and Nanog) [22,23,24]. Recently, the clinical significance of tumor-specific markers, such as CEA, CA125, and HE4 (better than epithelial-specific markers: EPCAM and MUC1) for CTCs isolation before and after adjuvant chemotherapy was shown [25]. Confirming the presence of CTCs in the blood of cancer patients and determining the cells’ phenotype have been indicated to be of diagnostic importance [7,8,9,11,12,13,14,15,16,18,24,26].

Combining CTCs profiling with other biomarkers assessment currently used for diagnosis and monitoring of OC patients may help find new combinations of markers with improved sensitivity and specificity.

However, it needs to be emphasized that none of the markers is specific and sensitive enough to identify all types of CTCs, especially in ovarian cancer patients, where CTCs number is rather low and the cells present with high heterogeneity. In fact, the only FDA-approved CTCs detection platform—CellSearch, detects epithelial CTCs, expressing both epithelial cell adhesion molecule (EpCAM) and cytokeratin, and might miss CTCs undergoing epithelial-to-mesenchymal transition.

Thus, only sensitive diagnostic techniques based on detailed analysis of CTCs-specific genetic profiles might allow the identification and isolation of the cells. This in turn, should increase the chances of metastasis detection.

### 2.1. The Ever-Changing Phenotype of CTCs

**Figure 2:**
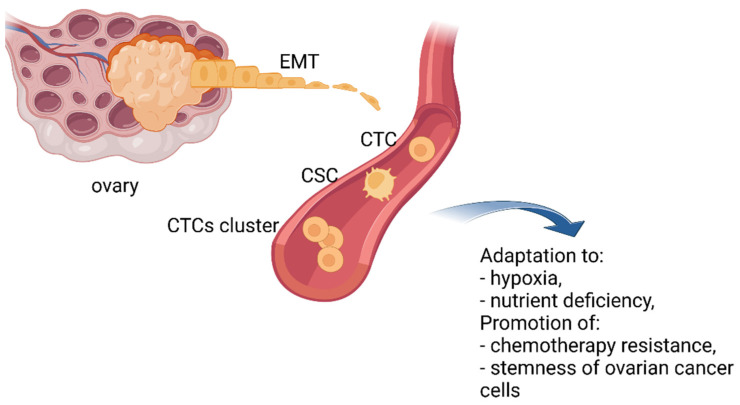
The ever-changing phenotype of CTCs. To increase their chances of survival, CTCs may change their phenotype from epithelial to mesenchymal, by clustering and/or acquiring cancer stem cell (CSC) properties. Created with https://biorender.com/, (accessed on 31 October 2022).

It has been demonstrated that even tumors without clinically confirmed metastasis can shed CTCs into the vascular or lymphatic system [12]. Still, a significant number of CTCs die before they reach a new niche. To increase their chances of survival and protect themselves from cell death CTCs may use different strategies. This includes changing their phenotype from epithelial to mesenchymal, clustering and/or acquiring cancer stem cell (CSC) features (Figure 2). CTCs are heterogeneous in nature. They consist of cell populations with different morphology, molecular characteristics, metastatic potential, and ability to survive chemotherapy. CTCs able to form metastases are known as invasive CTCs (iCTC) [11,19,21,22,25,27,28].

CTCs are believed to disseminate to distant sites thanks to epithelial–mesenchymal transition. This process includes a series of molecular, morphological, functional, and consequently, phenotypical changes of cells leading to the transition of polarized epithelial cells into mobile mesenchymal cells. Epithelial–mesenchymal transition may also generate hybrid phenotypes with an increased ability to survive in the circulation and adapt to various microenvironments [27].

A growing body of evidence demonstrates that EMT allows ovarian cancer cells to adapt to adverse conditions, such as hypoxia and nutrient deficiency, and promote chemotherapy resistance to therapeutic agents as well as activate the stemness of ovarian cancer cells [28]. Sharing some common features with cancer stem cells permits CTCs to increase their tumorigenicity and resist anoikis, chemo- and radiotherapy [29].

All this increases the heterogeneity of ovarian CTCs population and points to the significance of detailed molecular analysis of the cells’ expression profiles, especially in terms of their detection and clinical utility.

### 2.2. CTCs Clusters

Strong evidence suggests that CTCs can be organized in clusters. CTCs clusters may have up to 100-fold increased metastatic potential in comparison with the same number of isolated single CTCs. Clustering supports the collective migration of cells increasing their chances of survival, but also promotes specific changes such as stemness, drug resistance, and metastasis [29,30,31].

It has recently been proved that stemness and metastasis are promoted by specific changes in DNA methylation induced by the cells’ clustering. CTCs clustering leads to hypomethylation of binding sites for stemness and proliferation regulators, including OCT4, NANOG, SOX2, and SIN3A, and hypermethylation of Polycomb target genes [32]. 

Most cancers manifesting the presence of CTCs clusters are solid cancers and the clusters were detected in 16% to 75% of patients [31]. A higher number of clusters in patients’ blood was confirmed to be associated with shorter progression-free survival (PFS). This points to a possible link between CTCs clusters presence in peripheral blood and metastatic disease [15,31].

In ovarian cancer, CTCs clusters and their clinical relevance have not been extensively studied. Only a few studies demonstrate the presence of CTCs clusters in blood of OC patients and only a single research group provides information about their clinical significance [15,17,33]. This study describes CTCs clusters consisting of 2–30 cells. Such clusters are associated with platinum resistance, shorter time to progression (TTP), and PFS. Out of 24 OC patients with the primary disease and 30 patients with recurrences, CTCs were detected in 98.1%. Nevertheless, in women with the primary disease median counts of single CTCs and CTCs clusters were 4 and 1, and in those patients with recurrences, median counts were 3 and 1, respectively. Even though CTCs presence did not correlate with tumor stage and serum CA125 level, still CTCs counts ≥3 as well as CTCs clusters positivity correlated with platinum resistance and shortened overall survival in patients with recurrent disease. In the case of two patients CTCs isolation was followed by a successful in vitro culture. The results of ex vivo experiments indicated that CTCs can be more sensitive to anticancer drugs and proliferated more rapidly than established cell lines [15].

CTCs clusters in OC patients were also identified by Pearl et al. They demonstrated the invasive CTCs (iCTCs) isolated by functional cell adhesion matrix (CAM) uptake followed by microscopy and flow cytometry analysis using antibodies against epithelial/tumor antigens and negative selection with antibodies against hematopoietic lineage markers. These iCTCs tended to be heterogeneous in size and exhibited solitary cells and clusters. The changes in iCTCs and CA125 levels as well as changes in the intervals associated with no evidence of disease were noted. Additionally, an increased number of iCTCs (79.5%) was showed to be more sensitive than the increased CA125 level (67.6%) when it comes to predicting progressive disease (PD) or relapse. Finally iCTCs, but not CA125, preceded changes in the clinical status from PD to no evidence of disease during and after chemotherapy [17]. Thus, iCTCs in OC patients may help to predict the disease outcome and therapeutic responsiveness.

The presence of CTCs clusters isolated with the ALS CellCelector™ in ovarian cancers was also confirmed with liquid biopsy [33]. However, the authors of this study do not provide any information regarding the biology and/or clinical relevance of detected CTCs clusters.

Therefore, the development of efficient and reliable methods of CTCs clusters identification, together with cohort studies are needed to determine their suitability for clinical use.

### 2.3. CTCs and Cancer Stem Cells

In ovarian cancer, tumor cells are known to display cancer stem cells (CSCs) features such as self-renewal, differentiation, and tumorigenicity. CSCs are believed to support tumor growth and metastasis [29]. Due to the fact that they are also resistant to anoikis, CSCs may easily spread and survive within the lymphatic and vascular systems where they are considered to be stem CTCs [34,35,36,37,38].

Ovarian CSCs are characterized by the expression of specific markers. The best described include: CD44, CD133, CD24, CD117, Nestin, Nanog, and Oct3/4, as well as ALDH1A1 and ABC transporters. These markers allow CSCs detection and indicate tumor invasiveness, chemoresistance, and poor prognosis [29,33,38,39,40,41,42,43,44].

Some ovarian CSCs markers were reported to correlate with distinct metastasis via the haematogenous route. Recently, CD44 variant 6 was demonstrated to be a central player in the development of distant metastasis in parenchymal organs. A high number of CD44v6-positive ovarian cancer cells was associated with a high rate of distant metastasis at the time of diagnosis and distant metastasis-free survival varied significantly between CD44v6-high and -low patients [45]. Distant metastasis was also linked with the stem cell regulatory factor—EGFL6. EGFL6 induces cell division and migration of ALDH-positive ovarian CSCs, consequently promoting tumor growth and metastasis. Silencing of EGFL6 expression proved effective in reducing the haematogenous spread of ovarian cancer cells [46]. Thus, both CD44v6 and EGFL6 are involved in distant metastatic relapse and could be predictive biomarkers for distant parenchymal metastasis as well as a novel therapeutic target [45,46]. Their inhibition, in a similar way to blocking signal transduction pathways active in ovarian CSCs (e.g. Wnt, Hedgehog Notch, PI3K/PTEN/AKT) [47,48,49,50], seems to be a promising treatment alternative that should help overcome therapy resistance and reduce the mortality of ovarian cancer patients.

## 3. The Role of CTCs in Haematogenous Metastasis of Ovarian Cancer

In the 1980s, a series of studies documented that peritoneovenous shunting – a procedure that allows the peritoneal fluid to be returned from the peritoneal cavity into veins, does not significantly increase distant metastasis [10]. Conclusions based on those findings seem however far-fetched, as they did not take into consideration the overall well-being of patients who in most cases died within the next few months.

The first well-designed experiment proving the spread of OC via the bloodstream was performed by Pradeep and coworkers. Using a parabiosis model, where the skin of mice was fused surgically from the shoulder to the hip joint, they demonstrated that tumor cells can scatter in the bloodstream but eventually exit the circulatory system and enter the omentum [51].

Metastatic tumor cells are believed to seed to sites of favorable local microenvironment. Only CTCs that survived and crossed the physical barrier of the endothelium may seed the distant organ. At the molecular level, CTCs adhesion is a complex process, including cell-cell interactions between receptors located on CTCs and specific ligands located on the surface of endothelial membranes of organs. Inflammatory chemokines released by cells found in the pre-metastatic microenvironment interact with chemokine receptors expressed by CTCs and allow their targeted migration. In addition, chemokines may exert other functions, such as promoting tumor cell proliferation, angiogenesis, and immune system suppression [52].

Ovarian cancer CTCs may interact with the omentum via the HER3 receptor, whose presence is reported in 41–67.5% of ovarian cancer cases [53]. HER3 signaling plays an important role both in the development of ovarian cancer and its chemoresistance. Since the receptor’s expression can be upregulated by chemotherapy, HER3 is said to be associated with shorter survival time [54]. This, in turn, makes it a suitable biomarker candidate with therapeutic potential to stop ovarian cancer progression.

One other study points to the role of the chemokine receptor type 4 (CXCR4), which seems to determine the pro-invasive features of ovarian tumor cells [55]. Blocking the receptor with its antagonist—AMD3100 or specific anti-CXCR4 shRNA has caused inhibition of metastasis in animal models. Such response was the result of reduced levels of active Src, ERKs, inhibition of epithelial–mesenchymal transition (EMT), and blocking of hematogenous ovarian cancer dissemination by decreasing the number of circulating tumor cells [55].

Metastasis of OC may also be driven by signaling based on other receptor-ligand axes. Latest research implies the involvement of CCL5 and its receptors (CCR1, CCR3, and CCR5) as well as CCL20-CCR6, CCL25–CCR9, and CCL18. Pathways involving these signaling agents were previously reported to regulate ovarian cancer cells proliferation, mobility, epithelial–mesenchymal transition, and stem cell properties [52,56,57,58,59,60,61,62]. However, the role of these chemokines in CTCs invasion and its downstream-signaling pathways remain elusive. 

Hematogenous metastasis is also likely to be associated with the activation of the p90RSK family of serine/threonine kinases acting downstream of the RAS-ERK/MAPK pathway. Silencing of RSK1 and RSK2 isoforms abolished metastatic engraftment of ovarian cancer cells in the peritoneum and inhibited lung colonization after intravenous injection of cancer cells and hematogenous metastasis from subcutaneous xenografts. Both isoforms direct ovarian cancer cells in metastatic sites by regulating cell adhesion and invasion, probably through the activation of transcription and translation of factor YB-1, transcription of the FN1 gene, and translation of the TGF-β1 mRNA [63].

**Figure 3:**
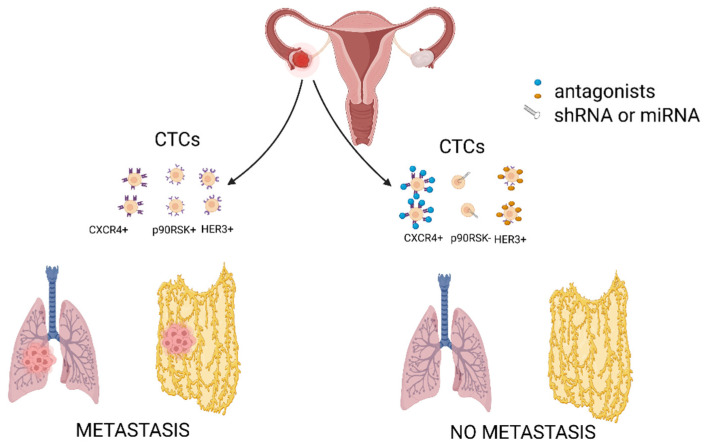
Ovarian CTCs may attach to the omentum or cause metastasis elsewhere. Pro-invasive features of ovarian tumor cells are determined via specific receptors, such as CXCR4 and p90RSK. Blocking of these targets may inhibit ovarian cancer metastasis. Created with https://biorender.com/, (accessed on 31 October 2022).

Ovarian CTCs may attach to the omentum or move on and establish metastases elsewhere (Figure 3). The metastatic pattern and organ-specificity of ovarian cancer have been documented by Coffman et al. [64]. They proved that intravenous injection of high-grade serous ovarian cancer cells resulted in the formation of intra-ovarian metastatic disease in mice. However, ovarian cancer cells were driven to the omentum only in the presence of the ovaries; this unique tropism for the peritoneal cavity is lost with oophorectomy. Moreover, the rate of metastasis to an otherwise healthy ovary was similar to rates of metastasis to the liver and lungs, even though these last two organs filter high volumes of intravenous cells during circulation and thus are exposed to many more tumor cells. This clearly points to a tropism of OC cells toward the ovary and its potential to grow within this organ [64].

Still, in OC patients, there are several other distant metastatic sites reported by oncologists. They include pleura and lymph nodes and, in rare cases, CNS, eye, skin, breast, bones, heart, central airways, rare intra-abdominal tissues, placenta, and specific lymph nodes [65].

In the hematogenous route of OC metastasis, cancer cells first invade the lymphovascular space (LVSI—lymphovascular space invasion) and then transit in blood or lymphatic vessels. Molecular profiling allows identifying some important molecules involved in this route. The analysis of different miRNAs expression between LVSI-positive and LVSI-negative ovarian cancer tissues and their association with bevacizumab response revealed that miR-25 expression correlates with a better PFS and OS in ovarian cancer. Thus, patients with low miR-25 expression and high miR-142 expression could benefit from bevacizumab treatment [66].

Furthermore, the analysis of OC transcriptome profiles with available information on LVSI status showed that primary tumors with increased risk of hematogenous and lymphatic metastasis highly express genes such as *POSTN, LUM, THBS2, COL3A1, COL5A1, COL5A2, FAP1,* and *FBN1*. All these genes are related to the extracellular matrix and extensive stromal activation [67].

Ovarian cancer cells may also enter the vasculature through the pelvic lymph nodes and via the left subclavian vein. This route of entry might contribute to the number of tumor cells found in the bloodstream of ovarian cancer patients at diagnosis [68].

## 4. Clinical Relevance of CTCs in Ovarian Cancer

Haematogenous spread of cancer cells in ovarian carcinomas is believed to be a rather rare event. Still, this phenomenon is well documented [7,8,9,11,12,13,14,15,16,17,18,19,20,21,23,24,26,32,43,50,69,70,71], and the presence of CTCs in blood was recently shown to correlate with tumor stage, presence of ascites, tumor debulking, disease recurrence, shorter OS and PFS [7,18,23,24,43,50,69].

The clinical significance of CTCs in ovarian cancer is presented in Table 1.

Changes in CTCs counts have been associated with the response to treatment. CTCs numbers correlate with overall tumor severity, and their status assessed before the start of systemic therapy is linked to clinical outcomes, such as shorter PFS and OS [12,14,15].

Banys-Paluchowski et al. provide data on the matter by showing that CTCs status evaluated prior to the start of systemic therapy correlates with the clinical outcomes predicting shorter OS and PFS [12]. However, another research group reports that CTCs counts do not correlate with the PFS of newly diagnosed EOC patients. Instead, they point to a correlation between CTCs cluster positivity and diminished OS of patients with recurrent disease and chemoresistance. Yet, this study was a proof of concept regarding a novel CTCs identification method with a limited number of patients [15].

Furthermore, Poveda et al. demonstrated the correlation of CTCs rate and PFS and OS of patients taking part in a phase III study of doxorubicin with trabectedin vs. doxorubicin alone in relapsed ovarian cancer. The results again confirm that elevated levels of CTCs prior to treatment increase the risk of progression and death of ovarian cancer patients. Specifically, the presence of CTCs (2 or more per blood sample) may be related to an unfavorable prognosis in recurrent ovarian cancer [16].

Positive CTCs status, independent of the time point of blood sampling, was also noted to be linked with shorter OS. The patients with persistent elevated CTCs counts ≥2 at baseline, and follow-up had shorter PFS and OS compared with patients with <2 CTCs [16,26]. Similarly, previously untreated patients with advanced OC and high CTCs counts ≥3 prior to chemotherapy had a significantly shorter PFS compared with patients with <3 CTCs. In turn, postoperative CTCs were more frequently detected in patients with lymph node involvement than in patients without it (100% vs. 30.0%). The cells’ presence seems to be associated with lower PFS rates in women with advanced stages of ovarian cancer [42].

A number of studies have also evaluated the utility of CTCs as a biomarker of chemotherapy response in OC [7,11,12,18,43,69,71]. It has been demonstrated that chemotherapy leads to a rapid decline in CTCs count. The overall CTCs number may decrease over time at a linear rate of 0.1 cells per month during the treatment [15].

In contrast, platinum-resistant OC patients were characterized by significantly higher CTCs counts compared to platinum-sensitive patients [7,17]. CTCs evaluation at primary diagnosis and after platinum-based chemotherapy showed that CTCs can be detected in 19% of patients before and in 27% after the treatment. CTCs counts were confirmed to be positively correlated with a shorter OS before surgery and after chemotherapy [7,43]. In addition, during the follow-up studies, the increase in CTCs number was more informative in predicting the disease progression or relapse than CA125 [9,17].

An important aspect of CTCs utility in terms of monitoring the disease is the changing phenotype of the circulating cells. Platinum-based chemotherapy may be followed by an increase in the incidence of epithelial-to-mesenchymal transition, which suggests that it promotes EMT-like phenotype formation. It is thus concluded that the occurrence of this particular subpopulation of EMT-like CTCs, rather than epithelial CTCs, might serve as a biomarker to identify patients at high risk of an unfavorable disease outcome. Therapy resistance, in this case, is suggested to be the consequence of clonal tumor evolution [18].

This hypothesis is supported by results published by Yang et al. In the study, CTCs counts, including those undergoing EMT, and clinical data were used to develop a predictive nomogram, which evaluates the risk triage of ovarian cancer recurrence. The group claims that routinely performed CTCs counts fitted to the nomogram model may also aid clinical decision making, especially in terms of early interventions for ovarian cancer patients [49].

Recently the percentage of the mesenchymal CTCs subgroup (M-CTC) has been proven to have better predictive value than both CA125 and other biomarkers of OC, including CA199, AFP, CEA, and HE4 [44]. They demonstrated that the cut-off value for a positive test was 5 for CTCs and 0.3 for mesenchymal CTCs. PFS and OC survival curves were significantly different when stratified by CTCs counts and M-CTCs percentage. Thus, the evaluation of CTCs populations may have a great prediction value for ovarian cancer prognosis, including chemoresistance and survival [44].

Other research groups, who attempted to examine the potential of CTCs to monitor EOC patients’ treatment, confirm the hypothesis that a well-designed CTCs assay has greater sensitivity than a standard serum CA125 measurement. What is more, for a number of patients undergoing standard taxol/carboplatin therapy, serial CTCs counts demonstrate better treatment responsiveness predictive ability than CA125 analysis. It is also claimed that especially the detection of iCTCs, along with a standard clinical evaluation, has the potential to provide better prognostic information on cancer metastasis in the early stages of the disease [9,17].

In the aforementioned studies, the isolation of iCTCs relied on the use of the cell adhesion matrix (CAM). Specifically, this enabled confirmation of the cells’ presence in 85.3% of analyzed patients, even in the early stages of EOC. iCTCs counts were correlated with tumor stage, debulking, and platinum sensitivity. Although the study includes a limited number of patients, the results still prove that iCTCs have the potential to become a suitable prognostic factor of metastasis [9,17].

Similar conclusions regarding CA125 were drawn by Kim et al., who assessed the relationships between this antigen and CTCs and the clinical outcome of the disease. It was reported that CTCs counts were better associated with treatment response and recurrence than CA125 levels [42].

Another interesting case is a study that demonstrated patients who were CTC-positive but did not show an increase in CA125 levels. This phenomenon was explained by the dissemination of cancer through the hematogenous route but not accompanied by peritoneal spread [22]. Zhang et al. support this rationale by stating that, in contrast to the well-known assumption that hematogenous metastasis occurs at a late stage of tumor development, this type of ovarian cancer spread, in fact, may occur earlier than peritoneal metastasis. Such conclusions were also drawn based on CTCs occurrence and evaluation of CA125 levels found in EOC [47]. Moreover, the published data suggest that the prognostic value of CTCs is independent of clinicopathological factors such as tumor type and grade, race, age, or platinum therapy status. It is concluded that the evaluation of CTCs may offer clinicians a more reliable method to predict cancer aggressiveness earlier [22].

CTCs’ clinical utility seems to rely greatly on molecular profiling of the isolated cells. For example, evaluation of gene expression followed by immunofluorescent staining proved to be useful in identifying a novel CTCs biomarker—PPIC. Experiments of quantification of PPIC expression in CTCs may help identify patients who are at risk of ovarian cancer recurrence, and for whom therapeutic strategy may need to be adjusted [7].

Another recent study by Zuo et al. attempted to correlate the clinical features of OC patients with the presence of CTCs and measurements of miR-181a. The results of the experiments indicate that quantification of miR-181a expression in CTCs may be an alternative method for early diagnosis and be of prognostic value [48].

Other researchers focus on the expression of markers, including EpCAM, MUC-1, HER-2, and WT1, and claim that such CTCs’ gene expression profiling makes it possible to predict the likelihood of chemotherapy resistance and evaluate prognosis as well as the potential to develop novel molecular targets for specific biological therapies. Zhang et al. also prove that using this methodology, CTCs may be detected in the blood of patients in all stages of EOC disease, while Aktas et al. and Wang et al. document that the occurrence of CTCs significantly correlates with shorter OS before surgery but also after chemotherapy [43,47,71].

Other studies document that quantification of ERCC1 (excision repair cross-complementation group 1) or cyclophilin 3 expression in iCTCs might be used to monitor platinum resistance and post-therapeutic outcome of ovarian cancer patients [7,23]. Those conclusions were based on the fact that platinum-based chemotherapy potentially selects ERCC1- and cyclophilin 3-positive CTCs [7,23].

Despite so many studies documenting the significance of circulating tumor cells in cancer progression, some research groups still find it unlikely for CTCs to become a diagnostic biomarker in ovarian cancers. Doubts may be the consequence of either insufficient support in the collected research data or simply the assumption that the nowadays available assays for cell enrichment are simply not suitable enough to become part of the standard procedures [11,50].

Moreover, the available data documenting the clinical utility of CTCs is very difficult to interpret and compare. Studies show no consistency in the process of patients’ enrollment, the amount of blood analyzed, or a consensus on the type of methodology applied for CTCs isolation and identification. Thus, much more effort should be made to develop better validation and uniform methodology for CTCs detection. This process also includes optimization of targeting CTCs surface markers to identify the cells more accurately. Perhaps in the case of ovarian cancer, more attention should also be drawn to CTCs clusters, which have been proven to be mediators of metastasis.

Most studies presented in the review indicate that CTCs detected in the blood of OC patients may have clinical significance. Even though their use as markers for ovarian cancer screening is still limited, CTCs can help in cancer diagnosis and evaluation of the disease prognosis. Moreover, since blood sampling is minimally invasive and allows serial analysis, CTCs provide a tool for therapy response monitoring and/or treatment modification, especially in the context of minimal residual disease as well as disease relapse monitoring.

Taking everything into consideration, a standardized methodology for CTCs and CTCs cluster detection and characterization should ensure high sensitivity and reproducibility of an assay that could become a clinically reliable blood test for predicting outcomes of women with ovarian cancer.

## 5. Conclusions

Identification of ovarian cancer cells and their dissemination is crucial for disease detection, monitoring of its progression, and treatment. Metastasis is strongly dependent on the molecular characteristics of invasive cancer cells. Thus, detailed analysis and understanding of the mechanisms driving OC metastasis are needed to improve patients’ survival. These mechanisms may lead to the personalization of the treatment, particularly the development of drugs and/or therapies blocking cancer cell dissemination.

The data supporting the clinical utility of CTCs in metastatic OC is very difficult to interpret and compare. The results of the up-to-date published research show no consistency in the process of patients’ enrollment, the amount of blood analyzed, or a consensus on the type of methodology applied for CTCs isolation and identification. Thus, much more effort should be made to develop better validation and uniform methodology for cell detection. Even though CTCs use as markers for ovarian cancer screening is still limited, they can support cancer diagnosis and evaluation of the disease prognosis.

## Figures and Tables

**Table 1:** Detection methods of ovarian CTCs and their clinical significance.

Author and Year of the Study	Patients Number	Blood Amount	CTCs Detection Method	CTCs Clinical Significance
Marth C. et al., 2002 [50]	90	40 mL	Microbeads coated with MOC-31 antibody.	CTCs were detected in 12% of patients.
			Enrichment with magnetic beads coupled with EGP-2 antibody.	CTCs rate varied between 10 and 150 tumor cells per 106 MNC.
Fan et al., 2009 [69]	66	5–20 mL	Ficoll density gradient centrifugation followed by cell invasion assay that enriches and identifies tumor cells with a cell adhesion matrix (Vita-Assay™).	CTCs were detected in 60.6% of patients.
				-10% in the early stage;
				-73.1% in the late stage.
				CTCs significantly correlated with decreased disease-free survival.
Aktas B. et al., 2011 [43]	122	10 mL	Immunomagnetically enriched tumor cells with antibody mixture (anti-GA 73.3 and anti-MUC1 antibodies).	Before surgery, CTCs were detected in 19% of patients.
			Analysis of tumor-associated mRNA performed by multiplex PCR for: HER2, MUC1, and GA 733-2.	After chemotherapy CTCs were detected in 27% of patients.
				CTCs positivity significantly correlated with shorter overall survival before surgery and after chemotherapy.
Poveda A. et al., 2011 [16]	216	10 mL	CellSearch system.	CTCs were detected in 51.4% of patients.
			CTCs identified as EpCAM+, cytokeratin+, CD45−, and positive for the nuclear stain.	Prior to the start of therapy, ≥ 2 CTCs were identified in 14.4% of patients.
				Patients with ≥2 CTCs prior to therapy had a significantly higher risk for progression and death.
				Patients with elevated baseline CTCs had a significantly higher risk of progression and death, respectively.
Pearl et al., 2014 [17]	129	2–20 mL	Cell adhesion matrix (CAM)-based functional cell enrichment and identification platform.	1.2% sensitivity, 95.1% specificity, and 77.8% positive predictive value (PPV) of iCTCs in detecting patients with stage I and II EOC malignancy.
			iCTCs identified as epithelial (Epi+)-positive and hematopoietic lineage (HL-)-negative when analyzed by flow cytometry and fluorescent microscopy imaging.	83% sensitivity and 97.3% PPV of iCTCs in detecting all stages of EOC malignancy.
Pearl M. et al., 2015 [9]	123	2–20 mL	Cell adhesion matrix (CAM)-based platform to isolate invasive CTCs (iCTCs).	iCTCs were detected in 85.3% of patients.
				-Positive predictive value (PPV) of iCTCs was 90%,
			iCTCs identified as epithelial (Epi+)-positive and hematopoietic lineage (HL-)-negative when analyzed by flow cytometry and fluorescent microscopy imaging,	-Negative predictive values (NPV) of iCTCs was 80.6%.
				Increases in iCTCs (79.5%) were more sensitive than increases in CA125 (67.6%) to predict progressive disease or relapse.
Kolostova K. et al., 2015 [22]	118	8 mL	MetaCell: size-based enrichment based on filtration.	CTCs were detected in 65.2% of patients.
			CTCs identified as cells with: (i) nuclear size ≥10 μm), (ii) irregular nuclear contour, (iii) visible cytoplasm, (iv) prominent nucleoli, (v) high nuclear-cytoplasmic ratio, (vi) proliferating, (vii) growing in 3D layers.	CTCs correlated with the presence of ascites, peritoneal carcinomatosis, and residual disease.
Blassl et al., 2016 [24]	10	5 mL	AdnaTest OvarianCancerSelect.	CTCs presence correlated with decreased overall survival.
			AdnaTest EMT-1/StemCellDetect.	CTCs with epithelial–mesenchymal-transition (EMT) or stem-like traits were pointed to be involved in metastatic progression and recurrence.
Chebouti et al., 2017 [18]	91	5 mL	AdnaTest OvarianCancer Detect.	Detection rate for epithelial CTCs was 18%.
			AdnaTest EMT-1 Detect.	Detection rate for EMT-like CTCs was 30%.
			Analysis of EpCAM, Muc-1, and CA125 and the EMT-associated transcripts: PI3Kα, Akt-2, and Twist.	PI3K+ EMT-like CTCs, in combination with epithelial CTCs, indicated decreased OS for FIGO I-III patients with residual tumor burden after surgery.
				Epithelial CTCs alone significantly correlated with decreased PFS and OS.
Chebouti et al., 2017 [23]	65	10 mL	AdnaTest Ovarian Cancer.	ERCC1^+^CTCs were detected in 15% of patients at primary diagnosis and in 12% after chemotherapy.
				ERCC1^+^CTCs after chemotherapy correlated with platinum resistance and reduced PFS and OS.
			Tumor-associated transcripts: EpCAM, MUC-1, and CA-125. ERCC1 was investigated by RT-PCR.	ERCC1^+^CTCs persistence indicated poor post-therapeutic outcome.
Lee M. et al., 2017 [15]	54	10 mL	Biotin-doped.	CTCs were detected in 98.1% of cases.
			Ppy-deposited microfluidic system with streptavidin.	Newly diagnosed patients’ median counts of single CTCs and CTC clusters were 4 and 1, respectively.
			Antibodies mixture directed against: EpCAM, TROP-2, EGFR, vimentin, and N-cadherin.	In primary and recurrent disease, median counts of CTCs clusters were 1 and 1, respectively.
			CTCs identified as EpCAM-positive and DAPI-positive, and CD45-negative cells.	In newly diagnosed patients with CTCs counts ≥ 3, PFS was significantly shorter. CTCs clusters positivity correlated with platinum resistance.
Lou E. et al., 2018 [11]	29	7.5 mL	Positive selection with magnetic beads conjugated to an anti-EpCAM antibody.	CTCs were detected in 17.2% of patients.
			CTC enumeration with DAPI, anti-CD45, and an anti-cytokeratin cocktail ( CK8, CK18, and CK19).	CTCs correlated with higher stage (FIGO stage III or IV) of tumor.
			CTCs identified as EpCAM-positive, CK-positive, DAPI-positive, and CD45-negative by the morphology of a single intact carcinoma cell (no cell clusters identified).	
Zhang X. et al., 2018 [47]	109	5 mL	Magnetic separation with beads coated with EpCAM, HER2, and MUC1 antibodies.	CTCs were detected in blood of 90% of newly diagnosed patients:
				- Average CTCs number: 264 (range 0–1929);
			RT-PCR analysis of EpCAM, HER2, and MUC1 expression.	- CTCs detected in 82%, 85%, 91%, and 100% of cases at stages I, II, III, and IV, respectively.
				CTCs were detected in 91% of patients after the treatment:
				- Average CTCs number: 314 (range 0–1822).
				Expression of EpCAM and HER in CTCs was correlated with resistance to chemotherapy.
				Expression of EpCAM in CTCs before the treatment was correlated with overall survival.
Kim M. et al., 2019 [42]	30	5 mL	Tapered-slit filter (TSF) platform.	Postoperative CTCs were more frequently detected in women with lymph node involvement: 100% vs. 30.0%.
			CTCs defined as (DAPI)-positive, (CD45)-negative, CK 9-positive, and EpCAM-positive, and using morphological criteria: higher nucleus-to-cytoplasm ratio, larger size, and higher degree of irregularity than observed in the background blood cell.	
Banys-Paluchowski M. et al., 2020 [12]	43	7.5 mL	CellSearch™ system (magnetic separation with beads coated with EpCAM).	Positive rate of CTCs: 27%.
				CTCs status before the start of systemic therapy correlated with clinical outcome.
			CTCs stained with several antibodies.	
Zuo Li et al., 2021 [48]	30	7 mL	Magnetic separation with beads coated with EpCAM.	Expression level of miR181 in CTCs was related to:
				- The stage of OC (in stages III and IV significantly higher than in stages I and II);
			miR181a expression determined by RT-PCR.	- The presence of lymphatic metastasis.
Obermayr E. et al., 2021 [7]	105	25 mL	Gradient centrifugation.	CTCs were detected in 24.5% of patients before the treatment. CTCs were detected in 20.4% of the patients after adjuvant treatment (follow-up patients).
			PPIC expression determined by IF and RT-PCR.	CTCs in follow-up patients were correlated with:
				- Age;
				- Resistance to platinum-based chemotherapy;
				- FIGO stage at borderline significance.
				Patients with PPIC-positive CTCs were characterized by significantly shorter disease-free survival than PPIC-negative patients (median PFS 11 vs. 21 months) and shorter overall survival.
				Presence of CTCs in patients after chemotherapy was associated with:
				- Increased mortality;
				- Higher risk of recurrence;
				- Increased mortality after 5 survived years.
Yang J. et al., 2021 [49]	181	5 mL	Nanofiltration technology.	CTC counts: 8.70 ± 5.69
				- M-CTC/total CTCs percentage: 0.24 ± 0.19;
			Epithelial E-CTCs (EpCAM, CK8/18/19), mesenchymal M-CTCs (vimentin, Twist), and epithelial/mesenchymal hybrid CTCs identified by RNA-In Situ hybridization (RNA-ISH) method.	- E-CTC/total CTCs percentage: 0.57 ± 0.25;
				- Hybrids/total CTCs percentage: 0.19 ± 0.11.
				Increase in recurrence rate:
				- CTCs ≥ 5–1.98-fold increase;
				- CTCs < 5–1.24-fold increase;
				- M-CTC < 0.1–1.43-fold increase.
Cheng H. et al., 2021 [20]	20	5 mL	Negative selection of leukocytes with immunomagnetic beads (anti-CD45).	CTCs were detected in 95.0% of patients.
			The cell size, quantified immunostaining intensity of CA125 and HE4, and ploidy of Chr8.	Total number of CTCs: 8.5 cells.
Ma et al., 2021 [44]	156	5 mL	Can Patrol^TM^ technique followed by RNA-ISH with probes for mesenchymal molecules (Vimentin and Twist) and epithelial cell adhesion molecules (CK8/18/19 and EpCAM).	CTC counts and M-CTC percentage provided significantly great prediction values for clinical stages, platinum resistance, and survival.
Wang et al., 2022 [71]	160	5 ml	Immunomagnetic beads targeting epithelial cell surface antigens (EpCAM and MUC1) and RT-PCR (detecting EpCAM, MUC1, and WT1).	Specificity of the CTCs detection was significantly higher than CA125 (92.2% vs. 82.2%).
				Detection rate of CTCs was higher than the positive rate of CA125 (74.5% vs. 58.2%) in early-stage patients.
				CTCs detection rate was significantly higher in patients with ascitic volume ≥500 mL.
				The detection rate of CTCs EpCAM+ and CTCs MUC1+ was significantly higher in chemo-resistant patients (26.3% vs. 11.9%; 26.4% vs. 13.4%).
				The median progression-free survival time for CTCs MUC1+ patients trended to be longer than CTCs MUC1− patients and overall survival was shorter in CTCs MUC1+ patients.

## References

[1] Siegel R.L., Miller K.D., Jemal A. (2020). Cancer Statistics, 2020. CA. Cancer J. Clin..

[2] Momenimovahed Z., Tiznobaik A., Taheri S., Salehiniya H. (2019). Ovarian Cancer in the World: Epidemiology and Risk Factors. Int. J. Womens Health.

[3] Howlader N., Noone A., Krapcho M., Miller D., Brest A., Yu M. SEER Cancer Statistics Review, 1975-2018, National Cancer Institute. Bethesda. https://seer.cancer.gov/csr/1975_2018/,.

[4] Rose P.G., Piver M.S., Tsukada Y., Lau T. (1989). Metastatic Patterns in Histologic Variants of Ovarian Cancer. An Autopsy Study. Cancer.

[5] Güth U., Arndt V., Stadlmann S., Huang D.J., Singer G. (2015). Epidemiology in Ovarian Carcinoma: Lessons from Autopsy. Gynecol. Oncol..

[6] Lengyel E. (2010). Ovarian Cancer Development and Metastasis. Am. J. Pathol..

[7] Obermayr E., Reiner A., Brandt B., Braicu E.I., Reinthaller A., Loverix L., Concin N., Woelber L., Mahner S., Sehouli J. (2021). The Long-Term Prognostic Significance of Circulating Tumor Cells in Ovarian Cancer—A Study of the OVCAD Consortium. Cancers.

[8] Kolostova K., Pinkas M., Jakabova A., Pospisilova E., Svobodova P., Spicka J., Cegan M., Matkowski R., Bobek V. (2016). Molecular Characterization of Circulating Tumor Cells in Ovarian Cancer. Am. J. Cancer Res..

[9] Pearl M.L., Dong H., Tulley S., Zhao Q., Golightly M., Zucker S., Chen W.-T. (2015). Treatment Monitoring of Patients with Epithelial Ovarian Cancer Using Invasive Circulating Tumor Cells (ICTCs). Gynecol. Oncol..

[10] Tarin D., Price J.E., Kettlewell M.G., Souter R.G., Vass A.C., Crossley B. (1984). Mechanisms of Human Tumor Metastasis Studied in Patients with Peritoneovenous Shunts. Cancer Res..

[11] Lou E., Vogel R.I., Teoh D., Hoostal S., Grad A., Gerber M., Monu M., Łukaszewski T., Deshpande J., Linden M.A. (2018). Assessment of Circulating Tumor Cells as a Predictive Biomarker of Histology in Women With Suspected Ovarian Cancer. Lab. Med..

[12] Banys-Paluchowski M., Fehm T., Neubauer H., Paluchowski P., Krawczyk N., Meier-Stiegen F., Wallach C., Kaczerowsky A., Gebauer G. (2020). Clinical Relevance of Circulating Tumor Cells in Ovarian, Fallopian Tube and Peritoneal Cancer. Arch. Gynecol. Obstet..

[13] Yousefi M., Dehghani S., Nosrati R., Ghanei M., Salmaninejad A., Rajaie S., Hasanzadeh M., Pasdar A. (2020). Current Insights into the Metastasis of Epithelial Ovarian Cancer—Hopes and Hurdles. Cell. Oncol..

[14] Zeng L., Liang X., Liu Q., Yang Z. (2017). The Predictive Value of Circulating Tumor Cells in Ovarian Cancer: A Meta Analysis. Int. J. Gynecol. Cancer.

[15] Lee M., Kim E.J., Cho Y., Kim S., Chung H.H., Park N.H., Song Y.-S. (2017). Predictive Value of Circulating Tumor Cells (CTCs) Captured by Microfluidic Device in Patients with Epithelial Ovarian Cancer. Gynecol. Oncol..

[16] Poveda A., Kaye S.B., McCormack R., Wang S., Parekh T., Ricci D., Lebedinsky C.A., Tercero J.C., Zintl P., Monk B.J. (2011). Circulating Tumor Cells Predict Progression Free Survival and Overall Survival in Patients with Relapsed/Recurrent Advanced Ovarian Cancer. Gynecol. Oncol..

[17] Pearl M.L., Zhao Q., Yang J., Dong H., Tulley S., Zhang Q., Golightly M., Zucker S., Chen W.-T. (2014). Prognostic Analysis of Invasive Circulating Tumor Cells (ICTCs) in Epithelial Ovarian Cancer. Gynecol. Oncol..

[18] Chebouti I., Kasimir-Bauer S., Buderath P., Wimberger P., Hauch S., Kimmig R., Kuhlmann J.D. (2017). EMT-like Circulating Tumor Cells in Ovarian Cancer Patients Are Enriched by Platinum-Based Chemotherapy. Oncotarget.

[19] Bregenzer M.E., Horst E.N., Mehta P., Novak C.M., Repetto T., Snyder C.S., Mehta G. (2019). Tumor Modeling Maintains Diverse Pathology in Vitro. Ann. Transl. Med..

[20] Cheng H., Wang S., Luan W., Ye X., Dou S., Tang Z., Zhu H., Lin P.P., Li Y., Cui H. (2021). Combined Detection and Subclass Characteristics Analysis of CTCs and CTECs by SE-IFISH in Ovarian Cancer. Chin. J. Cancer Res..

[21] Bankó P., Lee S.Y., Nagygyörgy V., Zrínyi M., Chae C.H., Cho D.H., Telekes A. (2019). Technologies for Circulating Tumor Cell Separation from Whole Blood. J. Hematol. Oncol..

[22] Kolostova K., Matkowski R., Jędryka M., Soter K., Cegan M., Pinkas M., Jakabova A., Pavlasek J., Spicka J., Bobek V. (2015). The Added Value of Circulating Tumor Cells Examination in Ovarian Cancer Staging. Am. J. Cancer Res..

[23] Chebouti I., Kuhlmann J.D., Buderath P., Weber S., Wimberger P., Bokeloh Y., Hauch S., Kimmig R., Kasimir-Bauer S. (2017). ERCC1-Expressing Circulating Tumor Cells as a Potential Diagnostic Tool for Monitoring Response to Platinum-Based Chemotherapy and for Predicting Post-Therapeutic Outcome of Ovarian Cancer. Oncotarget.

[24] Blassl C., Kuhlmann J.D., Webers A., Wimberger P., Fehm T., Neubauer H. (2016). Gene Expression Profiling of Single Circulating Tumor Cells in Ovarian Cancer—Establishment of a Multi-Marker Gene Panel. Mol. Oncol..

[25] Yousefi M., Rajaie S., Keyvani V., Bolandi S., Hasanzadeh M., Pasdar A. (2021). Clinical Significance of Circulating Tumor Cell Related Markers in Patients with Epithelial Ovarian Cancer before and after Adjuvant Chemotherapy. Sci. Rep..

[26] Asante D.-B., Calapre L., Ziman M., Meniawy T.M., Gray E.S. (2020). Liquid Biopsy in Ovarian Cancer Using Circulating Tumor DNA and Cells: Ready for Prime Time?. Cancer Lett..

[27] Genna A., Vanwynsberghe A.M., Villard A.V., Pottier C., Ancel J., Polette M., Gilles C. (2020). EMT-Associated Heterogeneity in Circulating Tumor Cells: Sticky Friends on the Road to Metastasis. Cancers.

[28] Loret N., Denys H., Tummers P., Berx G. (2019). The Role of Epithelial-to-Mesenchymal Plasticity in Ovarian Cancer Progression and Therapy Resistance. Cancers.

[29] Thankamony A.P., Saxena K., Murali R., Jolly M.K., Nair R. (2020). Cancer Stem Cell Plasticity—A Deadly Deal. Front. Mol. Biosci..

[30] Schuster E., Taftaf R., Reduzzi C., Albert M.K., Romero-Calvo I., Liu H. (2021). Better Together: Circulating Tumor Cell Clustering in Metastatic Cancer. Trends Cancer.

[31] Amintas S., Bedel A., Moreau-Gaudry F., Boutin J., Buscail L., Merlio J.-P., Vendrely V., Dabernat S., Buscail E. (2020). Circulating Tumor Cell Clusters: United We Stand Divided We Fall. Int. J. Mol. Sci..

[32] Gkountela S., Castro-Giner F., Szczerba B.M., Vetter M., Landin J., Scherrer R., Krol I., Scheidmann M.C., Beisel C., Stirnimann C.U. (2019). Circulating Tumor Cell Clustering Shapes DNA Methylation to Enable Metastasis Seeding. Cell.

[33] Nelep C., Eberhardt J. (2018). Automated Rare Single Cell Picking with the ALS Cellcelector^TM^. Cytom. Part A.

[34] Virant-Klun I., Zech N., Rožman P., Vogler A., Cvjetičanin B., Klemenc P., Maličev E., Meden-Vrtovec H. (2008). Putative Stem Cells with an Embryonic Character Isolated from the Ovarian Surface Epithelium of Women with No Naturally Present Follicles and Oocytes. Differentiation.

[35] Parte S., Bhartiya D., Telang J., Daithankar V., Salvi V., Zaveri K., Hinduja I. (2011). Detection, Characterization, and Spontaneous Differentiation In Vitro of Very Small Embryonic-Like Putative Stem Cells in Adult Mammalian Ovary. Stem Cells Dev..

[36] Liao J., Qian F., Tchabo N., Mhawech-Fauceglia P., Beck A., Qian Z., Wang X., Huss W.J., Lele S.B., Morrison C.D. (2014). Ovarian Cancer Spheroid Cells with Stem Cell-like Properties Contribute to Tumor Generation, Metastasis and Chemotherapy Resistance through Hypoxia-Resistant Metabolism. PLoS ONE.

[37] Keyvani V., Farshchian M., Esmaeili S.-A., Yari H., Moghbeli M., Nezhad S.-R.K., Abbaszadegan M.R. (2019). Ovarian Cancer Stem Cells and Targeted Therapy. J. Ovarian Res..

[38] Auersperg N. (2013). The Stem-Cell Profile of Ovarian Surface Epithelium Is Reproduced in the Oviductal Fimbriae, with Increased Stem-Cell Marker Density in Distal Parts of the Fimbriae. Int. J. Gynecol. Pathol..

[39] Bapat S.A., Mali A.M., Koppikar C.B., Kurrey N.K. (2005). Stem and Progenitor-Like Cells Contribute to the Aggressive Behavior of Human Epithelial Ovarian Cancer. Cancer Res..

[40] Hu L., McArthur C., Jaffe R.B. (2010). Ovarian Cancer Stem-like Side-Population Cells Are Tumourigenic and Chemoresistant. Br. J. Cancer.

[41] Muñoz-Galván S., Carnero A. (2020). Targeting Cancer Stem Cells to Overcome Therapy Resistance in Ovarian Cancer. Cells.

[42] Kim M., Suh D.H., Choi J.Y., Bu J., Kang Y.-T., Kim K., No J.H., Kim Y.B., Cho Y.-H. (2019). Post-Debulking Circulating Tumor Cell as a Poor Prognostic Marker in Advanced Stage Ovarian Cancer. Medicine.

[43] Aktas B., Kasimir-Bauer S., Heubner M., Kimmig R., Wimberger P. (2011). Molecular Profiling and Prognostic Relevance of Circulating Tumor Cells in the Blood of Ovarian Cancer Patients at Primary Diagnosis and after Platinum-Based Chemotherapy. Int. J. Gynecol. Cancer.

[44] Ma J., Yang J., Jin Y., Cheng S., Huang S., Zhang N., Wang Y. (2021). Artificial Intelligence Based on Blood Biomarkers Including CTCs Predicts Outcomes in Epithelial Ovarian Cancer: A Prospective Study. Onco. Targets. Ther..

[45] Motohara T., Fujimoto K., Tayama S., Narantuya D., Sakaguchi I., Tashiro H., Katabuchi H. (2016). CD44 Variant 6 as a Predictive Biomarker for Distant Metastasis in Patients With Epithelial Ovarian Cancer. Obstet. Gynecol..

[46] Bai S., Ingrfam P., Chen Y.-C., Deng N., Pearson A., Niknafs Y.S., O’Hayer P., Wang Y., Zhang Z.-Y., Boscolo E. (2016). EGFL6 Regulates the Asymmetric Division, Maintenance, and Metastasis of ALDH+ Ovarian Cancer Cells. Cancer Res..

[47] Zhang X., Li H., Yu X., Li S., Lei Z., Li C., Zhang Q., Han Q., Li Y., Zhang K. (2018). Analysis of Circulating Tumor Cells in Ovarian Cancer and Their Clinical Value as a Biomarker. Cell. Physiol. Biochem..

[48] Zuo L., Li X., Zhu H., Li A., Wang Y. (2021). Expression of MiR-181a in Circulating Tumor Cells of Ovarian Cancer and Its Clinical Application. ACS Omega.

[49] Yang J., Ma J., Jin Y., Cheng S., Huang S., Zhang N., Wang Y. (2021). Development and Validation for Prognostic Nomogram of Epithelial Ovarian Cancer Recurrence Based on Circulating Tumor Cells and Epithelial-Mesenchymal Transition. Sci. Rep..

[50] Marth C., Kisic J., Kaern J., Tropé C., Fodstad Ø. (2002). Circulating Tumor Cells in the Peripheral Blood and Bone Marrow of Patients with Ovarian Carcinoma Do Not Predict Prognosis. Cancer.

[51] Pradeep S., Kim S.W., Wu S.Y., Nishimura M., Chaluvally-Raghavan P., Miyake T., Pecot C.V., Kim S.-J., Choi H.J., Bischoff F.Z. (2014). Hematogenous Metastasis of Ovarian Cancer: Rethinking Mode of Spread. Cancer Cell.

[52] Izraely S., Witz I.P. (2021). Site-Specific Metastasis: A Cooperation between Cancer Cells and the Metastatic Microenvironment. Int. J. Cancer.

[53] Cîrstea A.E., Stepan A.E., Mărgăritescu C., Zăvoi R.E., Olimid D.A., Simionescu C.E. (2017). The Immunoexpression of EGFR, HER2 and HER3 in Malignant Serous Ovarian Tumors. Rom. J. Morphol. Embryol..

[54] Mizuno T., Kojima Y., Yonemori K., Yoshida H., Sugiura Y., Ohtake Y., Okuma H., Nishikawa T., Tanioka M., Sudo K. (2020). Neoadjuvant Chemotherapy Promotes the Expression of HER3 in Patients with Ovarian Cancer. Oncol. Lett..

[55] Figueras A., Alsina-Sanchís E., Lahiguera Á., Abreu M., Muinelo-Romay L., Moreno-Bueno G., Casanovas O., Graupera M., Matias-Guiu X., Vidal A. (2018). A Role for CXCR4 in Peritoneal and Hematogenous Ovarian Cancer Dissemination. Mol. Cancer Ther..

[56] Liu W., Wang W., Zhang N., Di W. (2020). The Role of CCL20-CCR6 Axis in Ovarian Cancer Metastasis. OncoTargets Ther..

[57] Zou W., Wicha M.S. (2015). Chemokines and Cellular Plasticity of Ovarian Cancer Stem Cells. Oncoscience.

[58] Long H., Xiang T., Qi W., Huang J., Chen J., He L., Liang Z., Guo B., Li Y., Xie R. (2015). CD133+ Ovarian Cancer Stem-like Cells Promote Non-Stem Cancer Cell Metastasis via CCL5 Induced Epithelial-Mesenchymal Transition. Oncotarget.

[59] Wang Q., Tang Y., Yu H., Yin Q., Li M., Shi L., Zhang W., Li D., Li L. (2016). CCL18 from Tumor-Cells Promotes Epithelial Ovarian Cancer Metastasis via MTOR Signaling Pathway. Mol. Carcinog..

[60] Johnson E.L., Singh R., Johnson-Holiday C.M., Grizzle W.E., Partridge E.E., Lillard J.W., Singh S. (2010). CCR9 Interactions Support Ovarian Cancer Cell Survival and Resistance to Cisplatin-Induced Apoptosis in a PI3K-Dependent and FAK-Independent Fashion. J. Ovarian Res..

[61] Cioffi M., D’Alterio C., Camerlingo R., Tirino V., Consales C., Riccio A., Ieranò C., Cecere S.C., Losito N.S., Greggi S. (2015). Identification of a Distinct Population of CD133(+)CXCR4(+) Cancer Stem Cells in Ovarian Cancer. Sci. Rep..

[62] Johnson E.L., Singh R., Singh S., Johnson-Holiday C.M., Grizzle W.E., Partridge E.E., Lillard J.W. (2010). CCL25-CCR9 Interaction Modulates Ovarian Cancer Cell Migration, Metalloproteinase Expression, and Invasion. World J. Surg. Oncol..

[63] Torchiaro E., Lorenzato A., Olivero M., Valdembri D., Gagliardi P.A., Gai M., Erriquez J., Serini G., Di Renzo M.F. (2016). Peritoneal and Hematogenous Metastases of Ovarian Cancer Cells Are Both Controlled by the P90RSK through a Self-Reinforcing Cell Autonomous Mechanism. Oncotarget.

[64] Coffman L.G., Burgos-Ojeda D., Wu R., Cho K., Bai S., Buckanovich R.J. (2016). New Models of Hematogenous Ovarian Cancer Metastasis Demonstrate Preferential Spread to the Ovary and a Requirement for the Ovary for Abdominal Dissemination. Transl. Res..

[65] Thomakos N., Diakosavvas M., Machairiotis N., Fasoulakis Z., Zarogoulidis P., Rodolakis A. (2019). Rare Distant Metastatic Disease of Ovarian and Peritoneal Carcinomatosis: A Review of the Literature. Cancers.

[66] Li J., Yue H., Li W., Zhu G., Zhu T., Chen R., Lu X. (2021). Bevacizumab Confers Significant Improvements in Survival for Ovarian Cancer Patients with Low MiR-25 Expression and High MiR-142 Expression. J. Ovarian Res..

[67] Yue H., Wang J., Chen R., Hou X., Li J., Lu X. (2019). Gene Signature Characteristic of Elevated Stromal Infiltration and Activation Is Associated with Increased Risk of Hematogenous and Lymphatic Metastasis in Serous Ovarian Cancer. BMC Cancer.

[68] Erdem B., Yüksel I.T., Peker N., Ulukent S.C., Aşıcıoğlu O., Özaydin I.Y., Ülker V., Akbayir O. (2018). Evaluation of Factors Affecting Lymph Node Metastasis in Clinical Stage I-II Epithelial Ovarian Cancer. Oncol. Res. Treat..

[69] Fan T., Zhao Q., Chen J.J., Chen W.-T., Pearl M.L. (2009). Clinical Significance of Circulating Tumor Cells Detected by an Invasion Assay in Peripheral Blood of Patients with Ovarian Cancer. Gynecol. Oncol..

[70] Andrusiewicz M., Szczerba A., Wołuń-Cholewa M., Warchoł W., Nowak-Markwitz E., Gąsiorowska E., Adamska K., Jankowska A. (2011). CGB and GNRH1 expression analysis as a method of tumor cells metastatic spread detection in patients with gynecological malignances. J. Transl. Med..

[71] Wang T., Gao Y., Wang X., Tian J., Li Y., Yu B., Huang L.H., Liang H., Irwin D.M., Ta H. (2022). Establishment of an optimized CTC detection model consisting of EpCAM, MUC1 and WT1 in epithelial ovarian cancer and its correlation with clinical characteristics. Chin. J. Cancer Res..

